# Supplementary figures and images for: Two components of body-image disturbance are differentially associated with distinct eating disorder characteristics in healthy young women
Source: PLoS One. 2022 Jan 12;17(1):e0262513. doi: 10.1371/journal.pone.0262513 (PMC8754315; doi:10.1371/journal.pone.0262513)

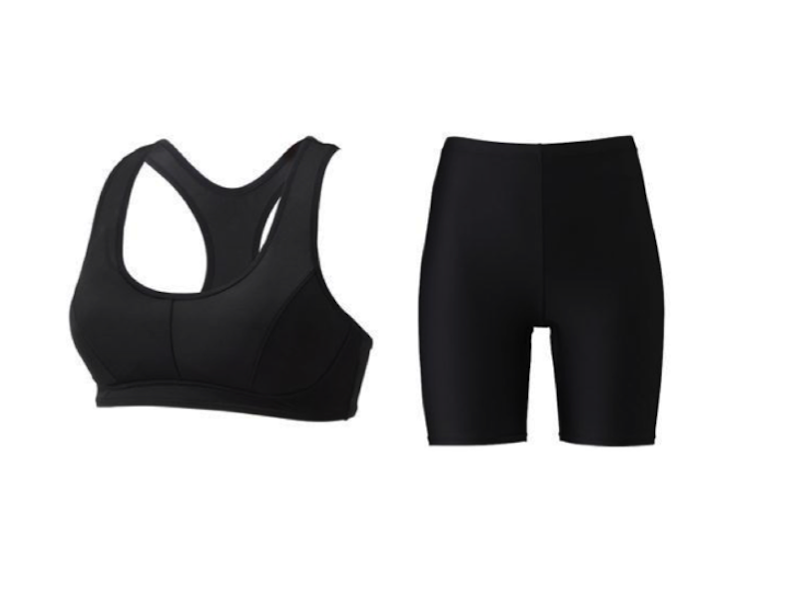

Supplement: S1 Fig — Participants were loaned a sports brassiere and leggings according to their height. If the clothes were not comfortable, we provided a medium-large-sized garment (the standard size was small-medium). (TIF) [file pone.0262513.s002.tif]
