# Supplementary material for: Two components of body-image disturbance are differentially associated with distinct eating disorder characteristics in healthy young women
Source: PLoS One. 2022 Jan 12;17(1):e0262513. doi: 10.1371/journal.pone.0262513 (PMC8754315; doi:10.1371/journal.pone.0262513)
Supplement: Attachment — Submitted filename: Response to Reviewers.docx [file pone.0262513.s004.docx]

Dear Editor, *PLOS ONE,*

Thank you for giving us the opportunity to submit our revised manuscript entitled “Two components of body-image disturbance are differentially associated with distinct eating disorder characteristics in healthy young women” to *PLOS ONE.* We appreciate your comments and those of the reviewers, and have revised the manuscript accordingly.

Our responses to the comments by the reviewers (reviewers’ comments in *italics*) are as follows:

===================================================

Response to Reviewer 1

===================================================

**General**

*Thank you for carefully considering my feedback comments. All my comments have been sufficiently addressed by the authors. All the best with your research.*

**Reply**

Thank you for carefully reviewing our manuscript again. We appreciate your insightful comments.

===================================================

Response to Reviewer 2

===================================================

**General**

*It is clear that authors have taken time and made an effort to improve the manuscript, and have done a great job in giving attention to all comments. I reread the entire manuscript first, and the improvements are impressive! The flow of the language is better, and descriptions have been more clearly stated, making the manuscript and the investigation an interesting article to read.*

**Reply**

Thank you for carefully reviewing our manuscript again and providing insightful suggestions and comments. We revised the manuscript according to the reviewer’s suggestions to enhance readability.

**Comment 1**

*When looking at the specific comments from the former review, there are just some minor remarks that you can consider to revise:*

*• I think your aim could be even more to the point if you include EDI-2 in your first sentence of the aim, p.7 line 149-151.*

**Reply**

Thank you for your suggestion. As the reviewer suggested, we included EDI-2 in the first sentence of the study aims part. The edited text reads as follows:

“The present study aimed to determine the relationships between two body-image-disturbance components (perceptual and affective components) and eating-disorder characteristics represented by EDI2 scores.” (page 7, lines 149-151)

**Comment 2**

*• Your reply to comment 22, p.24, line 477-482, could these results be linked to low variation in BMI in your study group? Where there any overweight or obese individuals in your study group?*

**Reply**

The BMI in our study ranged from 17.58 to 24.87 (i.e., there were no obese individuals [BMI > 25.0 kg/m^2^]). Although this BMI range is consistent with that of Japanese females in their twenties, it is slightly lower than that in other countries.

We believe that the variation in BMI in our study was sufficiently informative given its significant correlation with dissatisfaction toward one’s body (factor 2 of EDI2) and perceived–ideal discrepancy. In the revised manuscript, we have discussed the role of BMI in the relationship between perceived–ideal discrepancy and dissatisfaction toward one’s body, as follows:

“We also observed that BMI was strongly correlated with dissatisfaction toward one’s body (r[27] = 0.71, t = 5.22, p < 0.001) and perceived–ideal discrepancy (r[26] = 0.61, t =3.94, p < 0.001). However, the correlation between perceived–ideal discrepancy and dissatisfaction toward one’s body remained significant after controlling for BMI.” (page 24, lines 478-482)

The fact that all of the participants were underweight or had a normal BMI (17.58–24.87) has been cited as one of the limitations of the study, i.e., the questionable generalizability of our results, as pointed out by the reviewer in Comment 4. The lack of obese participants was not surprising because the prevalence of obesity in Japanese females in their twenties is only 8.9%. The mean BMI in the current study was similar to that in previous studies conducted in Japan, but lower than that in previous studies conducted in other countries. We have added the following sentences to the limitations:

“Second, our results have limited cross-national generalizability. The BMI of the participants in the current study was similar to that in previous body-image studies conducted in Japan (47, 48), but lower than that in studies conducted in Western countries (12, 13). The lack of obese individuals (BMI > 25.0 kg/m^2^) in the current study is explained by the low prevalence of obesity in Japanese females in their twenties (i.e., 8.9% according to a national survey conducted in 2019 by the Ministry of Health, Labor and Welfare). BMI is a critical factor in body-image studies because it is strongly correlated with concerns about body image (12, 27-29). The comparability of our findings with those of studies that recruited participants from different cultures is also limited by our use of the Japanese version of the EDI2, which has a culture-specific six-factor structure. However, the results for factor 2 may be directly comparable to those of other studies because this factor is primarily composed of body-dissatisfaction items from the original EDI2 subscale. In contrast, factor 3 is composed of items from four subscales of the original EDI2: ineffectiveness, interoceptive awareness, impulse regulation, and social insecurity. In addition, this study had a small sample, although it was based on a power analysis. Due to the small sample size, relationships between the two components of body-image disturbance and eating disorder characteristics with small effect sizes may have been missed. Biological and cultural factors reflected in the lower BMI range and structure of the EDI2 would have influenced our findings and limit the generalizability of the current study. International comparative studies including larger samples and wider ranges of BMI will be needed to confirm our results.” (pages 27-28, lines 547-569)

**Comment 3**

*Two comments that were not included in first review, and became apparent upon second review:*

*• In the method section, I could not find information on when EDI-2 was administered, before or after the experiment?*

**Reply**

We apologize for the poor description of the procedure. We administered the EDI-2 before the experiment to avoid any effects of the tasks on the questionnaire results. We have added the following description:

“After completing the EDI2 questionnaire, participants performed the actual-body and ideal-body tasks (Fig. 1).” (page 12, lines 240-241)

“The participants completed the EDI2 (20) before performing the actual-body and ideal-body tasks.” (page 15, lines 314-315)

**Comment 4**

*• One limitation worth mentioning is the limited sample size, could this have any implications for your results or generalizability?*

**Reply**

Thank you for your suggestion. As pointed out by the reviewer, the sample size was small; however, it was adequate to detect the relationships of interest according to the power analysis. However, we may not have detected relationships between the two components of body-image disturbance and eating-disorder characteristics with small effect sizes, which is one of the study limitations. The implications of the small sample size for generalizability are discussed in the limitations section. We have added the following sentences:

“Second, our results have limited cross-national generalizability. The BMI of the participants in the current study was similar to that in previous body-image studies conducted in Japan (47, 48), but lower than that in studies conducted in Western countries (12, 13). The lack of obese individuals (BMI > 25.0 kg/m^2^) in the current study is explained by the low prevalence of obesity in Japanese females in their twenties (i.e., 8.9% according to a national survey conducted in 2019 by the Ministry of Health, Labor and Welfare). BMI is a critical factor in body-image studies because it is strongly correlated with concerns about body image (12, 27-29). The comparability of our findings with those of studies that recruited participants from different cultures is also limited by our use of the Japanese version of the EDI2, which has a culture-specific six-factor structure. However, the results for factor 2 may be directly comparable to those of other studies because this factor is primarily composed of body-dissatisfaction items from the original EDI2 subscale. In contrast, factor 3 is composed of items from four subscales of the original EDI2: ineffectiveness, interoceptive awareness, impulse regulation, and social insecurity. In addition, this study had a small sample, although it was based on a power analysis. Due to the small sample size, relationships between the two components of body-image disturbance and eating disorder characteristics with small effect sizes may have been missed. Biological and cultural factors reflected in the lower BMI range and structure of the EDI2 would have influenced our findings and limit the generalizability of the current study. International comparative studies including larger samples and wider ranges of BMI will be needed to confirm our results.” (pages 27-28, lines 547-569)
